# Budesonide-Loaded Guar Gum Microspheres for Colon Delivery: Preparation, Characterization and *in Vitro/in Vivo* Evaluation

**DOI:** 10.3390/ijms16022693

**Published:** 2015-01-26

**Authors:** Ye Liu, Hong Zhou

**Affiliations:** Department of Gastroenterology Surgery, Renji Hospital, School of Medicine, Shanghai Jiao Tong University, Shanghai 200127, China; E-Mail: liuye@renji.com

**Keywords:** budesonide, colon delivery, microspheres, *in vitro* release, pharmacokinetics

## Abstract

A novel budesonide (BUD) colon delivery release system was developed by using a natural polysaccharide, guar gum. The rigidity of the microspheres was induced by a chemical cross-linking method utilizing glutaraldehyde as the cross-linker. The mean particle size of the microspheres prepared was found to be 15.21 ± 1.32 µm. The drug loading and entrapment efficiency of the formulation were 17.78% ± 2.31% and 81.6% ± 5.42%, respectively. The microspheres were spherical in shape with a smooth surface, and the size was uniform. The *in vitro* release profiles indicated that the release of BUD from the microspheres exhibited a sustained release behavior. The model that fitted best for BUD released from the microspheres was the Higuchi kinetic model with a correlation coefficient *r* = 0.9993. A similar phenomenon was also observed in a pharmacokinetic study. The prolongation of the half-life (*t*_1/2_), enhanced residence time (mean residence time, MRT) and decreased total clearance (CL) indicated that BUD microspheres could prolong the acting time of BUD *in vivo*. In addition, BUD guar gum microspheres are thought to have the potential to maintain BUD concentration within target ranges for a long time, decreasing the side effects caused by concentration fluctuation, ensuring the efficiency of treatment and improving patient compliance by reducing dosing frequency. None of the severe signs, like the appearance of epithelial necrosis and the sloughing of epithelial cells, were detected.

## 1. Introduction

Budesonide (BUD) is a glucocorticoid with high anti-inflammatory activity and low systemic effects due to high receptor affinity, selectivity and rapid diversion [1,2]. One of the most common clinical uses of BUD is in ulcerative colitis (UC) and Crohn’s disease (CD). As we all know, UC and CD are the two main types of idiopathic inflammatory bowel disease (IBD) [3]. However, the currently available medications, such as aminosalicylates, steroids and immunosuppressants, are limited [4]. For example, the use of BUD is associated with side effects, like nose irritation or burning, bleeding, upset stomach, *etc*. To achieve maximum therapeutic effect with a low risk of adverse effects, sustained released preparations are preferred [5]. An ideal system allows the delivery of adequate amounts of drug to the target site with adequate release kinetics.

Colon delivery of a therapeutic drug may reduce the systemic side effects and provide effective and safe therapy that may reduce the dose and duration of therapy when compared with the conventional treatment. However, various strategies have been used for targeting colon, such as pH-sensitive polymers, coating with biodegradable polymers, fabrication of pro-drugs, timed release systems, embedding in biodegradable matrices and hydrogels [6,7]. Polysaccharides, such as pectin, guar gum, chitosan and amylose, have extensively been studied and widely accepted for colon targeting [8]. The matrices of polysaccharides are assumed to remain intact in the physiological environment of stomach and small intestine, but once in the colon, they are acted upon by bacterial polysaccharases [9]. 

The bioadhesive and biodegradable property of guar gum make it the first choice for developing controlled and targeting drug delivery systems for colon [10]. Guar gum obtained from the seeds of *Cyamopsis tetragonolobus* consists of linear chains of (1→4)-β-d-mannopyranosyl units with α-d-galactopyranosyl units attached by (1→6) linkages [11]. It is hydrophilic in nature and swells in cold water, forming viscous colloidal dispersions or sols. This gelling property retards the release of the drug from the dosage form, as well as its susceptibility to degradation in the colonic environment.

In this study, a BUD colon delivery release system was developed by using a natural polysaccharide, guar gum. The system consists of cross-linked microspheres of guar gum, which release BUD locally in the physiological environment of the colon. In additional, *in vitro* and *in vivo* evaluations of the characterization, release, pharmacokinetics and pharmacodynamics were conducted in order to provide the pharmacokinetic parameters for the further study of BUD sustained-release preparations.

## 2. Results and Discussion

### 2.1. Physicochemical Characterization

**Figure 1:**
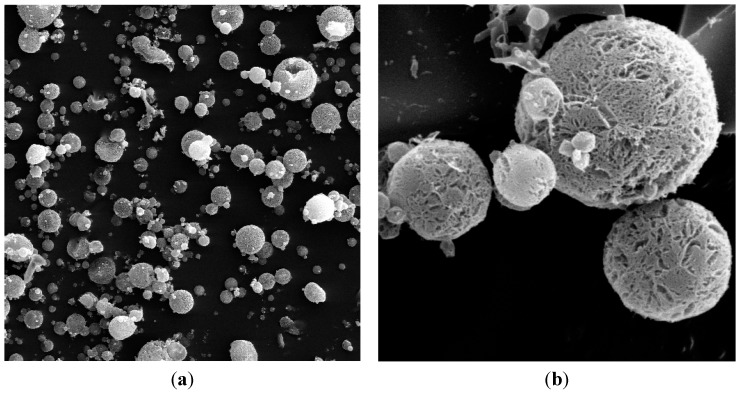
Scanning electron microscope photograph of budesonide (BUD) microspheres: (**a**) ×500; (**b**) ×2000.

Drugs that are used for the treatment of diseases associated with the colon require the passage of the formulation in an intact form through the stomach and small intestine and the release of the whole amount of drug in the colon. The conventional dosage form normally dissolves in the stomach or small intestine and gets absorbed from these regions of the GIT; thus, a much lesser amount of the drug reaches the colon. To obtain maximum therapeutic efficacy, it becomes necessary to deliver the agent to the target site in the optimal amount for the right period of time, thereby causing little toxicity and minimal side effects. Attempts have been made to achieve colon-specific drug delivery using polysaccharides. Wei *et al*. [12] have investigated pectin/ethyl cellulose film-coated tablets, whereas Krishnaiah *et al*. [13] have proposed matrix tablets of guar gum for colon-specific drug delivery. In the present investigation, cross-linked microspheres of guar gum were prepared for colon targeted delivery of BUD by the emulsification technique. The rigidity of the microspheres was induced by a chemical cross-linking method utilizing glutaraldehyde as the cross-linker. The effect of various process variables, such as stirring speed, stirring time, glutaraldehyde treatment and temperature, was analyzed in order to optimize the formulation. It was observed that these variables considerably influenced the shape, size, as well as the entrapment efficiency of formulations. Tween 80 was used for the purpose of the wetting of guar gum. The particle size of the cross-linked guar gum microspheres was determined using a laser diffraction particle size analyzer. The mean particle size of the prepared microspheres was found to be 15.21 ± 1.32 µm. The drug loading and entrapment efficiency of the formulation were 17.78% ± 2.31% and 81.6% ± 5.42%, respectively. As is shown in Figure 1, the surface morphology of BUD microspheres was observed by scanning electron microscopy (SEM). The microspheres were spherical in shape with a smooth surface, and the size was uniform and appropriate for oral administration.

### 2.2. In Vitro Release

**Figure 2:**
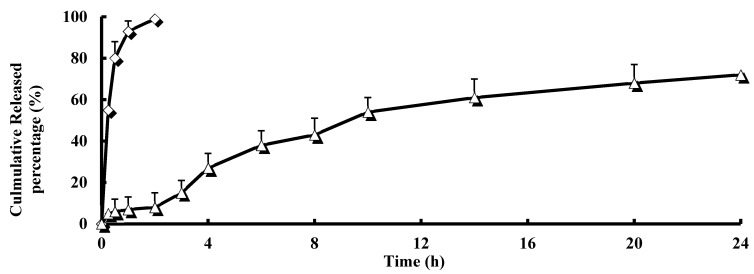
Drug release profiles of BUD microspheres (△) and suspensions (◊) in simulated gastric fluid (SGF) (pH 1.2) for 2 h (Phase I), followed by simulated intestinal fluid (SIF) (pH 6.8) for the remaining time (Phase II) (*n* = 5).

The cross-linked microspheres of guar gum were subjected to *in vitro* drug release rate studies in simulated gastric fluid (SGF) (pH 1.2) for 2 h and simulated intestinal fluid (SIF) (pH 7.5) for 22 h in order to investigate the capability of the formulation to withstand the physiological environment of the stomach and intestine. Figure 2 shows the *in vitro* release for BUD microspheres and suspensions, respectively. A very fast release behavior of BUD was observed in suspensions, whereas the cumulative release rate of BUD microspheres was much slower followed by a sustained release. In the suspension group, more than 90% of the drug was released in the first hour of the dissolution process in the simulated gastric medium. Almost 100% of BUD were released in the first two hours. In contrast, only 8% of BUD were released from microspheres in the first 2 h. BUD was gradually released from the microspheres as time lapsed (~40% released in 6 h, ~54% released in 10 h and ~72% released in 24 h), suggesting that BUD was well entrapped in the guar gum microspheres. It is a fact that as the guar gum comes in contact with the dissolution medium, it creates a viscous gel layer around itself, which controls the release of the entrapped drug. The initial release of the drug present on the surface was higher during the 2 h study, which could be due to the fact that there was no viscous gel layer around the particles, and this might have formed after 2 or 3 h, which controlled the further release of the drug. These results are concordant with the results of Krishnaiah *et al*. [14], who have used matrix and compression coated tablets of guar gum, respectively, for colon targeted delivery. Several models (zero-order, first-order, Higuchi and Weibull model) describing drug release from immediate and modified release dosage forms were adopted. The model that fit best for BUD released from the microspheres was the Higuchi kinetic model with a correlation coefficient *r* = 0.9993, revealing that BUD was released in a controlled manner from the microspheres.

### 2.3. Pharmacokinetic Studies

**Figure 3:**
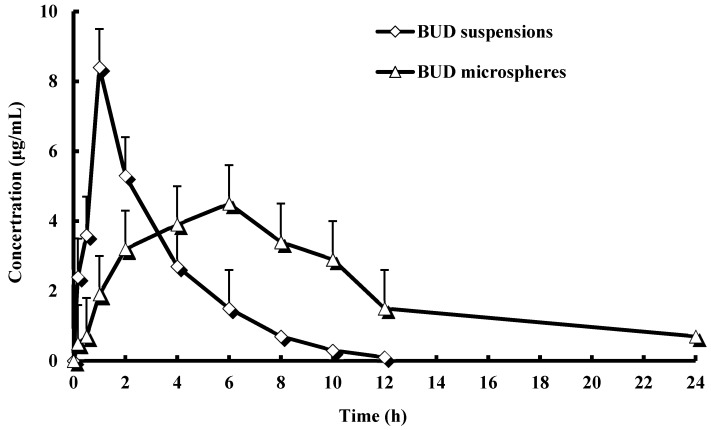
Mean plasma concentration time profiles of BUD after intragastric administration of a single 3.5 mg/kg dose of the suspension and microspheres to rats (each point represents the mean ± SD of six rats)

The profiles of BUD concentration in plasma *vs*. time are shown in Figure 3. Based on the analysis of the models and parameters with the DAS2.0 practical pharmacokinetics program, it was concluded that the *in vivo* pharmacokinetics of BUD microspheres could be described by a two-compartment model after intragastric administration. The pharmacokinetic parameters, such as area under the drug concentration-time curve values (AUC_0–∞_), mean residence time (MRT), clearance (CL) and biological half-life (t_1/2a_, t_1/2b_), are reported in Table 1. From the plasma profile, it was shown that the maximum concentration (*C*_max_) values for BUD suspensions and BUD microspheres were 8.41 and 4.57 μg/mL, respectively, and the time to reach maximum concentration (*T*_max_) values were 1 and 6 h, respectively. As shown in Table 1, the pharmacokinetic parameters of BUD microspheres in rats showed significant changes in comparison to those of the BUD suspensions. Guar gum microspheres provided higher AUC_0–∞_ (1.9-fold), MRT (3.98-fold), clearance half-life (*t*_1/2α_) (2.56-fold) and distribution half-life (*t*_1/2b_) (2.58-fold) compared to the BUD suspensions. The BUD microspheres also decreased CL and *C*_max_ compared to the BUD suspensions, respectively. The prolongation of the half-life (*t*_1/2_), enhanced residence time (MRT) and decreased total clearance (CL) indicated that BUD microspheres could prolong the acting time of BUD *in vivo*. The variation of AUC_0–∞_ from 35.63 ± 11.42 to 67.82 ± 21.32 µg·h/mL indicated that the BUD microspheres provided higher bioavailability than BUD suspensions. The Tmax was 6 h for BUD microspheres, while it was 1 h for BUD suspensions. This may be due to the fact that the drug release from the microspheres occurs after reaching the colon. On the other hand, drug release and absorption from BUD suspensions occurs in stomach, intestines and probably colon, as well. 

**Table 1:** Pharmacokinetic parameters of the two formulations. MRT, mean residence time; CL, clearance.

Parameter	Formulations	
	Suspensions	Microspheres
*t*_1/2α_ (h)	0.34 ± 0.12	0.87 ± 0.35 *
*t*_1/2β_ (h)	2.16 ± 1.03	5.58 ± 1.72 *
*C*_max_ (µg/mL)	8.41 ± 2.12	4.57 ± 1.34 *
AUC_0–t_ (µg·h/mL)	27.45 ± 9.61	50.74 ± 15.29 *
AUC_0–∞_ (µg·h/mL)	35.63 ± 11.42	67.82 ± 21.32 *
MRT (h)	1.16 ± 1.08	4.62 ± 1.32 *
CL (L/h)	3.21 ± 0.83	0.82 ± 0.12 *

* *p* < 0.05: BUD microspheres *vs*. BUD suspensions.

### 2.4. Evaluation of Colon Targeting

**Figure 4:**
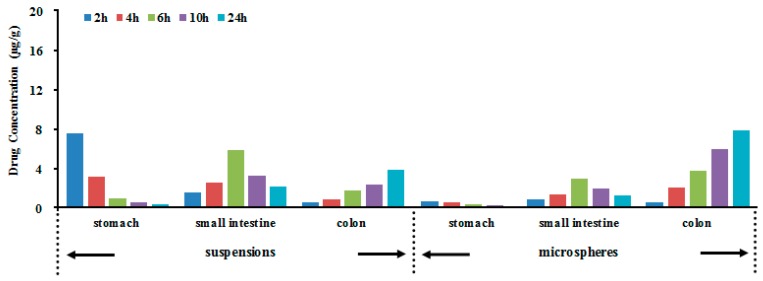
Distribution in tissue in rats following intragastric administration of a single 3.5 mg/kg dose of BUD suspensions and microspheres (each point represents the mean ± SD of six rats).

**Figure 5:**
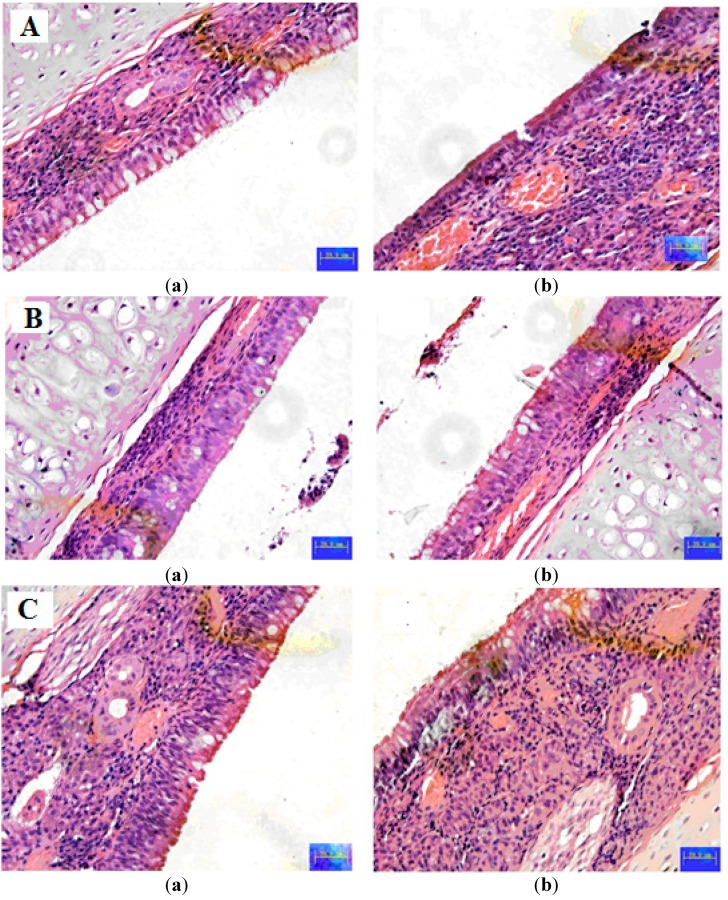
Histopathological studies of stomach (**A**) small intestine (**B**) and colon (**C**). (**a**) Saline and (**b**) BUD microspheres. Scale bar: 2 µm.

The *in vivo* biodistribution behavior of BUD after intragastric administration of the microspheres to rats was investigated with suspensions as a control. The amounts of drug distributed in unit mass of stomach and small intestine at various times were measured. Figure 4 presents the mean concentration-time profiles of BUD in unit mass of each organ in rats. The results showed that the maximum concentration of BUD, that is 7.5 µg/mL, was observed after 2 h in stomach after intragastric administration of BUD suspensions, that a small amount reached the small intestine, and that no drug was found in colon (Figure 4). Only a 3.8 µg/mL concentration of drug was determined in colon after 8 h. The BUD microspheres were found to be intact in the upper part of the GI tract. After 8 h of administration of BUD microspheres, the maximum percentage of drug was observed in the colon, and a negligible amount of drug was found in the stomach and a small amount in the small intestine. The total amount of drug accumulated in each organ within 24 h (AUC_0–t_) was calculated, and the results are shown in Table 2. The drug concentrations were significantly different in stomach, small intestine and colon between the microspheres and suspensions. The BUD AUC_0–t_ of the microspheres was 2.15-fold higher compared to suspensions in colon (*p* < 0.05). Hence, BUD guar gum microspheres are thought to have the potential to maintain the BUD concentration within target ranges for a long time, decreasing the side effects caused by concentration fluctuation, ensuring the efficiency of treatment and improving patient compliance by reducing dosing frequency. The histopathological examination of stomach, small intestine and colon was carried out for the detection of any damage to the tissue. The microphotographs were taken of stomach, small intestine and colon following incubation with microsphere formulations for more than 24 h (Figure 5). Saline was the control. None of the severe signs, like the appearance of epithelial necrosis and the sloughing of epithelial cells, were detected.

**Table 2:** The AUC_0–24 h_ of BUD in stomach, small intestine and colon after intragastric administration of suspensions and microspheres to rats (*n* = 5).

Formulation	Stomach	Small Intestine	Colon
BUD suspensions (µg·h/g)	23.63 ± 5.03	67.54 ± 19.51	54.81 ± 16.71
BUD microspheres (µg·h/g)	5.65 ± 1.38	35.26 ± 11.79	117.62 ± 21.82
Ratio ^a^	0.24	0.52	2.15 *

^a^ The ratio was AUC (BUD microspheres)/AUC (BUD suspensions); * *p* < 0.05: BUD microspheres *vs.* BUD suspensions.

## 3. Materials

BUD was from Hubei DKY Pharmaceutical Company (Wuhan, China, purity 99.9%, batch number: A1549). Glutaraldehyde, Tween-80 and methylene chloride were purchased from Sinopharm, Shanghai, China. All reagents were of high performance liquid chromatography (HPLC) (Sigma-Aldrich, Shanghai, China) grade, including acetonitrile and methanol. Other reagents were of analytical grade. Purified water from a Milli-Q system (Millipore, Bedford, MA, USA) was used throughout the experiment.

### 3.1. Microspheres Preparation

Guar gum microspheres were prepared by the emulsion cross-linking technique [15,16]. Briefly, BUD (25 mg) and guar gum (125 mg) were added to 5 mL of methylene chloride. After being completely dissolved, the solution was then slowly added to 2% Tween-80 aqueous solution, and then, the mixture was emulsified by using a propeller stirrer at 500 rpm for 45 min. Then, the 25% glutaraldehyde solution was slowly added to the emulsion system and cross-linked for 2 h, until the microspheres were solidified. The microspheres were collected by filtration through a 20 μm sieve, then washed three times with deionized water and dried in a vacuum desiccator for 48 h.

### 3.2. Physicochemical Characterization

#### 3.2.1. Particle Size Analysis

The particle size and size distribution were determined by photon correlation spectroscopy (PCS) using Malvern Zetasizer 2000 HS (Malvern Instruments, London, UK). The size measurement was performed in triplicate at room temperature, and results are expressed as the mean ± the standard deviation (SD).

#### 3.2.2. Scanning Electron Microscopy

The external morphology of microspheres was analyzed by scanning electron microscopy (SEM). A drop of microparticles was spread on a silicon wafer fixed with a sample holder for SEM and coated afterwards with gold using a gold sputter in a high vacuum evaporator and observed on SEM JEOL 7000F (Tokyo, Japan).

#### 3.2.3. Drug Loading

To determine the loading content of BUD (Budesonide) in microspheres, the microparticles were immersed in a methanol solution for 24 h, followed by sonication using a probe-type sonicator for 20 s at 70 W. The extract was filtered through a 0.45 μm syringe filter (Millipore), and the concentration of BD was determined by the high performance liquid chromatography (HPLC) method described below.

### 3.3. In Vitro Drug Release

The *in vitro* drug release studies were performed using a USP dissolution rate rest apparatus (paddle apparatus, 100 r/min, (37 ± 0.1) °C). Guar gum microspheres bearing BUD were suspended in simulated gastric fluid (SGF) pH 1.2 (900 mL) for 2 h. The dissolution media were then replaced with simulated intestinal fluid (SIF) pH 7.5. At predetermined time points of 0.25, 0.5, 1, 2, 3, 4, 6, 8, 10, 14, 20 and 24 h, 2 mL of dissolution media were withdrawn and precipitated before HPLC analysis. The supernatant (20 μL) was then directly injected into the HPLC system and analyzed for the released BUD. The BUD suspensions (0.5% HPMC (Hydroxypropyl methyl cellulose)) were investigated as a contrast.

### 3.4. Pharmacokinetic Studies

Twelve healthy Sprague Dawley (SD) rats (half males) were selected for the *in vivo* study, which was approved by the university’s committee on the ethical treatment of animals. The formulation of BUD microspheres was selected in order to study the *in vivo* performance of the preparation, on the basis of *in vitro* release studies. SD rats of similar weight were selected for *in vivo* studies, kept in well-spaced ventilated cages and maintained on a normal diet (soaked in water). The animals were divided into two groups of six animals each (with 3.5 mg/kg). The first group received the BUD suspension, which was prepared using 0.5% HPMC. The second group was given the formulation of guar gum microspheres. Blood samples (0.5 mL) were collected by heparinized tubes from the caudal vein at 10, 30 min and 1, 2, 4, 6, 8, 10, 12 and 24 h after intragastric administration. Blood was immediately processed to obtain plasma by centrifugation at 12,000 rpm for 10 min. Plasma samples were frozen and maintained at −70 °C for the upcoming analysis. Pharmacokinetic parameters were calculated against the plasma concentration-time data. The elimination half-life (*T*_1/2_) was determined by linear regression of the terminal portion of the plasma concentration-time data. The area under the plasma concentration-time curve from zero to the last measurable plasma concentration point (AUC_0–t_) was calculated by the linear trapezoidal method. Extrapolation to infinite time (AUC_0–∞_) was calculated as follows: AUC_0–∞_ = AUC_0–t_ + *C*_t_/ke, where *C*_t_ is the last measurable plasma concentration and ke is the terminal elimination rate constant. The results were expressed as the mean ± the standard deviation (SD).

### 3.5. Evaluation of Colon Targeting [17]

Sixty rats were used in the experiment to assess the effect of microspheres formulation on the intestinal biodistribution of BUD after intragastric administration. The rats were divided into two groups at random, and each of them was given a single 3.5 mg/kg dose of either the BUD microspheres or BUD suspensions. At 2, 4, 6, 10 and 24 h after drug administration, each animal (*n* = 6 for each time point) was euthanized, and stomach, small intestine and colon samples were collected. Tissue samples were washed in ice-cold saline, blotted with paper towel to remove excess fluid, weighed and stored at −70 °C until being assessed for drug concentration by HPLC. In addition, the organs at the time point of 24 hours (stomach, small intestine and colon) were pressed between filter pads, weighed and then fixed in 10% neutral formalin using standard techniques and stained with hematoxylin and eosin for histopathological examination. All tissue samples were examined and graded under light microscopy with 500× magnification.

### 3.6. Analysis Method

HPLC analysis was performed using a Dikma Diamonsil C18 (5 μm, 200 mm × 4.6 mm) on a Shimadzu LC-20A HPLC system (Shimadzu Co., Tokyo, Japan) with an ultraviolet detector at room temperature. The wavelength of the ultraviolet detector was set at 245 nm. Water and ethanol (57:43, *v*/*v*) were used as the mobile phase at a flow rate of 1 mL/min.

One hundred microliters of the plasma sample were transferred to a 10 mL plastic test tube together with 10 µL of I.S. solution (1 mg/mL triamcinolone). After vortex shaking for 30 seconds (Eppendorf, 5432 vortex mixer, Hamburg, Germany), 3 mL of ethyl acetate were added, and the mixture was vortexed for another 2 min. After centrifugation at 12,000 rpm for 10 min (Thermo IEC, Waltham, MA, USA), the upper organic layer was quantitatively transferred to a 10 mL glass tube and evaporated to dryness using an evaporator at 45 °C. The residue was reconstituted in 100 µL of the mobile phase and then vortex mixed. After centrifugation at 12,000 rpm for 5 min, a 20 µL aliquot of the solution was injected into the HPLC system for analysis.

Tissue samples were homogenized in a mixed solution of 200 µL PBS solution (pH = 6.0). Ten microliters of I.S. solution (1 mg/mL triamcinolone) were added to 200 µL of tissue samples and vortexed for 1 min. The drug and internal standard were then extracted into 3 mL of ethyl acetate by vortex mixing for 2 min. After centrifugation at 12,000 rpm for 10 min, the clear supernatant was removed and evaporated under a gentle stream of nitrogen. The residue was then dissolved by 100 µL of the mobile phase and centrifuged at 12,000 rpm for 5 min. Then, a 20 µL aliquot of the solution was injected into the HPLC system for analysis.

### 3.7. Statistical Analysis

All data are presented as the mean ± the standard deviation. Statistical analysis was performed using the Student’s *t*-test. Values of *p* < 0.05 were considered statistically significant.

## 4. Conclusions

In this study, a novel budesonide (BUD) colon delivery release system was developed by using a natural polysaccharide, guar gum. The microspheres were spherical in shape with a smooth surface, and the size was uniform and appropriate for oral administration. These *in vivo* studies revealed that the prepared BUD microspheres were effective at delivering BUD directly to colon in high concentrations, which would improve the prospects for a successful treatment outcome. Thus, in this study, a BUD microsphere formulation useful in the treatment of colon disease has been developed.
